# Supplementary figures and images for: New Brunswick’s mental health action plan: A quantitative exploration of program efficacy in children and youth using the Canadian Community Health Survey
Source: PLoS One. 2024 Jun 7;19(6):e0301008. doi: 10.1371/journal.pone.0301008 (PMC11161078; doi:10.1371/journal.pone.0301008)

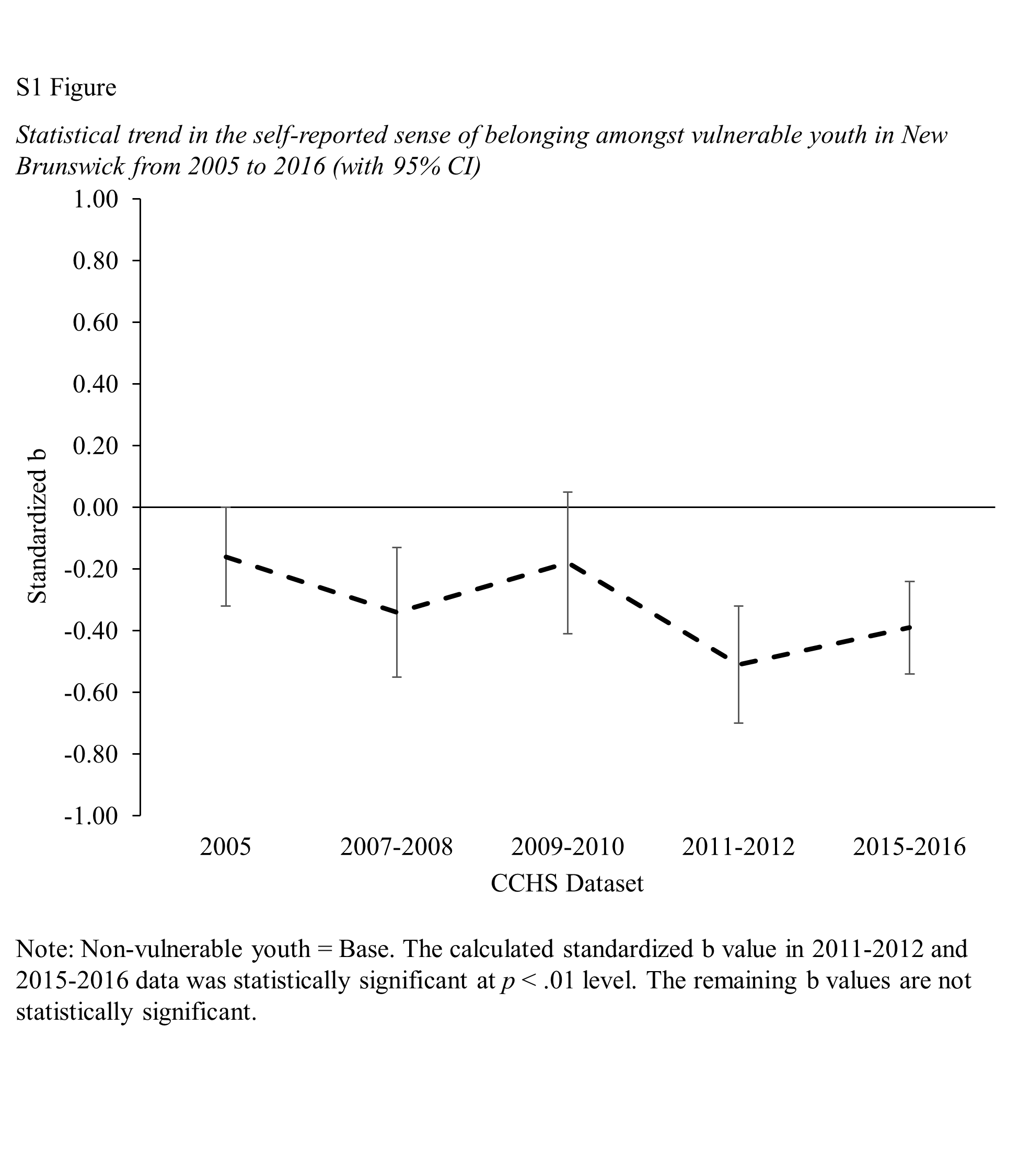

Supplement: S1 Fig — (TIF) [file pone.0301008.s001.tif]

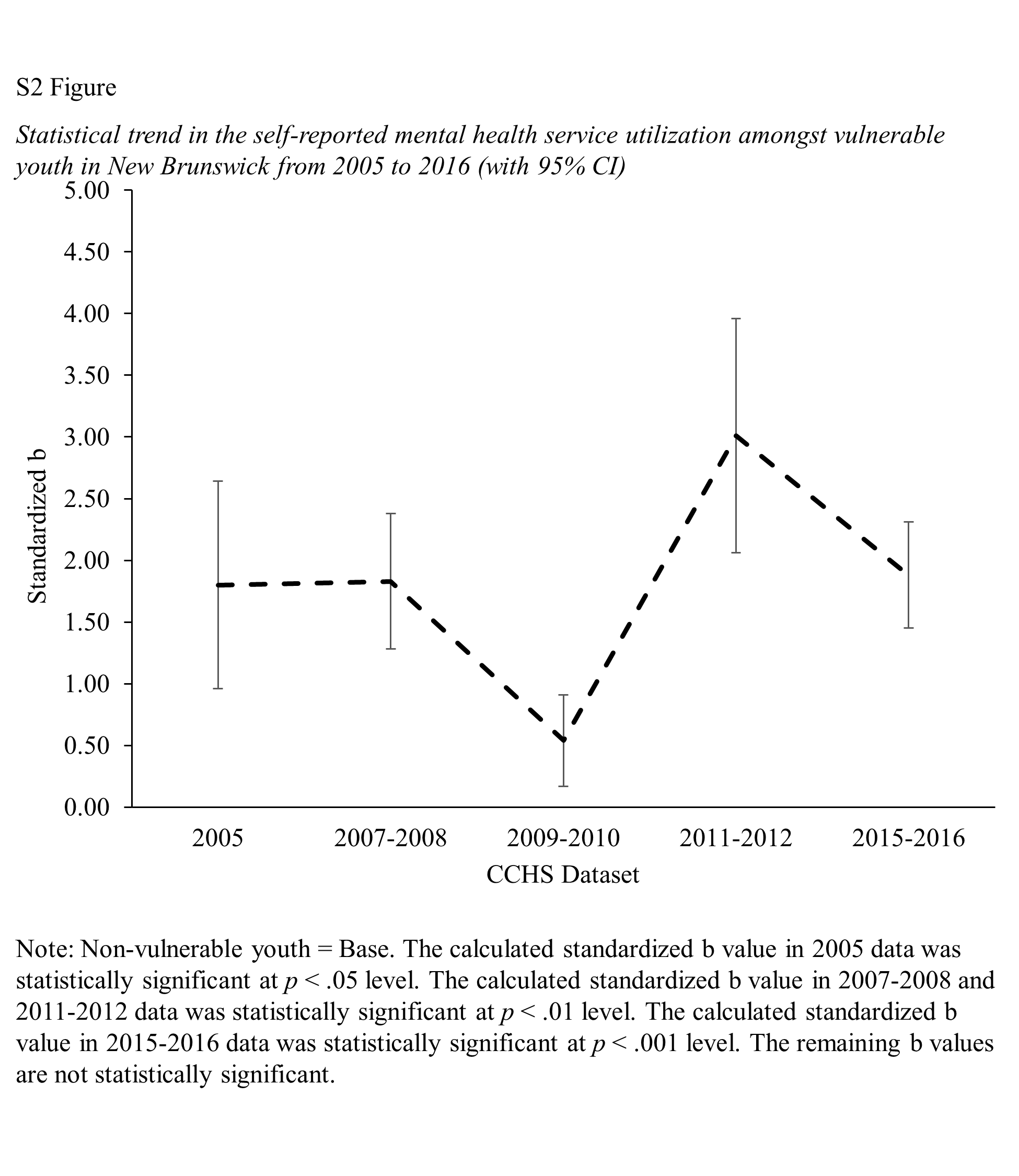

Supplement: S2 Fig — (TIF) [file pone.0301008.s002.tif]

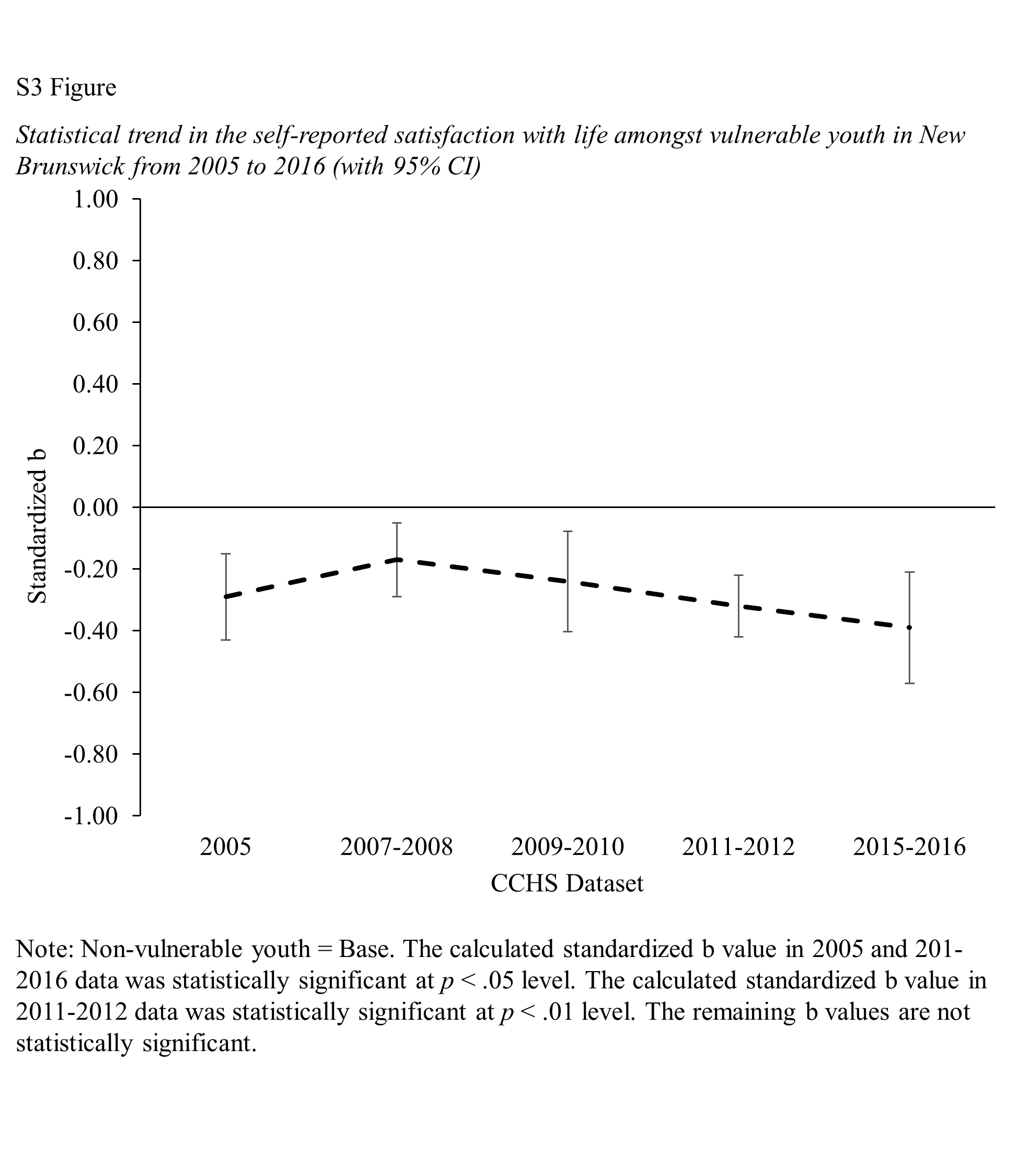

Supplement: S3 Fig — (TIF) [file pone.0301008.s003.tif]

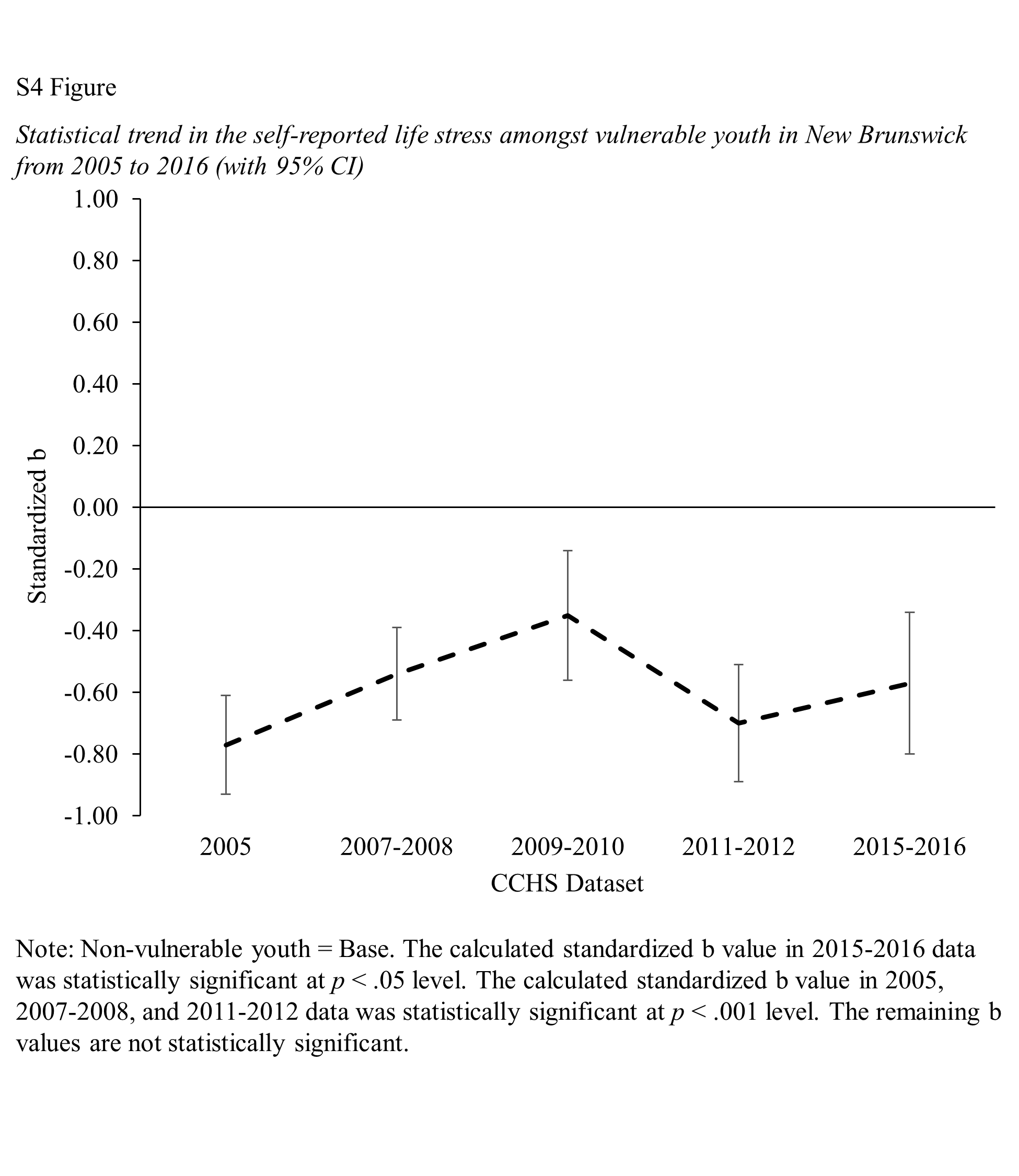

Supplement: S4 Fig — (TIF) [file pone.0301008.s004.tif]
