# Supplementary material for: New Brunswick’s mental health action plan: A quantitative exploration of program efficacy in children and youth using the Canadian Community Health Survey
Source: PLoS One. 2024 Jun 7;19(6):e0301008. doi: 10.1371/journal.pone.0301008 (PMC11161078; doi:10.1371/journal.pone.0301008)
Supplement: S1 Table — (DOCX) [file pone.0301008.s005.docx]

| **S1 Table** |  |  |  |  |  |
| --- | --- | --- | --- | --- | --- |
| *Descriptive statistics for covariates and outcomes by dataset* | | | | | |
|  | CCHS 2005 (N = 250) | CCHS 2007-2008 (N = 227) | CCHS 2009-2010 (N = 333) | CCHS 2011-2012 (N = 410) | CCHS 2015-2016 (N = 272) |
| *Sex* |  |  |  |  |  |
| Female | 48.40% | 44.10% | 47.70% | 51.50% | 51.50% |
| Male | 51.60% | 55.90% | 52.30% | 48.50% | 48.50% |
| *Visible Minority Status* |  |  |  |  |  |
| Non-Visible minority | 95.60% | 96.50% | 94.90% | 93.70% | 96.00% |
| Visible minority | 4.40% | 3.50% | 5.10% | 6.30% | 4.00% |
| *Marital Status* |  |  |  |  |  |
| Married/Common-Law | 2.00% | 2.60% | 1.50% | 1.70% | 0.70% |
| Widowed/Separated/Divorced | 0.00% | 0.00% | 0.00% | 0.00% | 0.00% |
| Single | 98.00% | 97.40% | 98.50% | 98.30% | 99.30% |
| *Dwelling Ownership* |  |  |  |  |  |
| Non-owner | 14.80% | 17.20% | 11.70% | 16.10% | 13.60% |
| Owner | 85.20% | 82.80% | 88.30% | 83.90% | 86.40% |
| Vulnerable Population Status |  |  |  |  |  |
| Non-vulnerable | 91.60% | 89.90% | 91.30% | 89.30% | 85.30% |
| Vulnerable | 8.40% | 10.10% | 8.70% | 10.70% | 14.70% |
| Income | 3.54/1.14 | 3.33/1.32 | 3.52/1.36 | 3.53/1.35 | 4.00/1.27 |
| Household Size | 3.56/0.89 | 3.44/0.93 | 3.66/0.93 | 3.55/0.88 | 3.91/0.89 |
| Self-rated Physical Health | 3.76/0.86 | 3.82/0.81 | 3.82/0.80 | 3.77/0.80 | 3.98/0.85 |
| Sense of Belonging | 2.87/0.89 | 2.77/0.84 | 3.08/0.74 | 2.97/0.71 | 3.11/0.66 |
| Mental Health Service Use | 0.28/1.41 | 0.41/1.60 | 0.44/1.74 | 0.71/2.33 | 0.61/2.23 |
| Satisfaction with Life | 4.32/0.58 | 4.39/0.64 | 4.49/0.62 | 4.47/0.59 | 4.53/0.59 |
| Self-rated Life Stress | 3.44/0.89 | 3.39/0.89 | 3.54/0.93 | 3.59/0.86 | 3.61/0.96 |
