# Supplementary material for: New Brunswick’s mental health action plan: A quantitative exploration of program efficacy in children and youth using the Canadian Community Health Survey
Source: PLoS One. 2024 Jun 7;19(6):e0301008. doi: 10.1371/journal.pone.0301008 (PMC11161078; doi:10.1371/journal.pone.0301008)
Supplement: S2 Table — (DOCX) [file pone.0301008.s006.docx]

| **S2 Table** | | |
| --- | --- | --- |
| *Overview of the New Brunswick Action Plan for Mental Health 2011-2018* | | |
| **Goals Identified** | **Initiatives Implemented** | **Summary & Status** |
| Goal 1: Improving the accessibility and delivery of services | Enhanced Provincial Diversion Model | Enabling offenders with mental health needs to receive meaningful and less intrusive intervention.  Implemented as of 2015 |
|  | Mental health training for correctional and probation officers | Implemented as of 2015 |
|  | Service network for children and youth with mental health needs | Implemented after 2015 progress report |
|  | Early Psychosis Program | Aimed to provide support and intervention for individuals experiencing first episodes of psychosis.  Implemented as of 2015 |
|  | Flexible Assertive Community Treatment services development | Systematic review for the development and implementation of Flexible Assertive Community Treatment services in health zones.  Implemented after 2015 progress report (New Brunswick Department of Health, 2021) |
|  | Centre of excellence for children and youth with complex mental health needs | Funding announced as of 2015 |
|  | New Brunswick Drug Plan | Improve drug accessibility for low-income earners.  Implemented as of 2015 |
|  | Social Assistance Adjustment | Rate increases and policy changes for provincial social assistance program.  Implemented as of 2015 |
|  |  |  |
|  |  |  |
| Goal 2: Supporting individuals with a recovery-based approach | Establishment of local and provincial recovery teams | Aimed to promote the implementation of the recovery model in mental health services via learning and training sessions.  Implemented as of 2015 |
|  | Training curriculum for physician and healthcare staff | Aimed to integrate mental health care in the primary healthcare setting.  Pilot sites implemented as of 2015 |
|  | Supportive Housing Network (SUN) | Coordinated effort to support homeless individuals in obtaining sustainable housing.  Implemented as of 2015 |
|  | Monitoring instruments | 2014 Primary Health Services Survey: Assessed the mental health services usage and experience, findings not disseminated in the progress reports.  2013 Mental Health Service Client Satisfaction Survey: Assessed client satisfaction in outpatient counselling services, the results are reported to be "largely positive." |
| Goal 3: Maintaining a person-centred approach | Supporting domestic violence outreach programs | 14 outreach programs supported.  Implemented as of 2015 |
|  | Environmental scan identifying mental health service needs for the First Nation population | Implemented as of 2015 |
|  | Creation of Aboriginal student counsellor position at St. Thomas University | Implemented as of 2015 |
|  | Creation of First Nations Counsellor Certificate program at the University of New Brunswick | Implemented as of 2015 |
|  |  |  |
|  | Youth Engagement Service program | Offered changes in rate and benefits structure for youth aged 16-18 for better support.  Implemented as of 2015 |
|  | Integrated service delivery model for youth with mental health needs | Implemented post-report in 2018 |
|  | Training curriculums for care workers and home support employees | Aimed to improve mental-health awareness amongst frontline workers.  Implemented as of 2015 |
| Goal 4: Alleviating social isolation in individuals with mental illness | "Your Recovery Journey" campaign | Intended to support individual recovery journeys via group sessions.  Implemented as of 2015 |
|  | Improve accessibility to the General Education Development and Workplace Essential Skills training in various provincial correctional facilities | Implemented as of 2015 |
|  | Career development and employment counselling services for individuals returning to the workforce | Implemented as of 2015 |
|  | Pre-Employability Services project | Introducing modifications in case management for clients with challenges affecting employability  Implemented as of 2015 |
|  | Overcoming Poverty Together: The New Brunswick Economic and Social Inclusion Plan | Roadmap for the province to move towards economic and social inclusion for all.  Implemented as of 2015 |
| Goal 5: Supporting the education and training of mental health professionals | Establish working group for comprehensive mental health education for students in health and social science programs | Implemented as of 2015 |
|  | Research funding for the Health Education Research Group at the University of New Brunswick | Aimed to support research related to the development of positive mental health communities.  Implemented as of 2015 |
|  | New bursaries to interns in psychology | Implemented as of 2015 |
|  | Mental health first aid training for frontline staffs | Offered mental health first aid training to all anglophone school districts, TeleCare 811, New Brunswick Community College and Collège communautaire du Nouveau-Brunswick  Implemented as of 2015 |
|  | Changing Minds awareness training | Training offered to frontline workers in health, social and educational fields.  Implemented as of 2015 |
| Goal 6: Reducing the stigma associated with mental illness | Positive mental health module for grade 9 and 10 students | Implemented as of 2015 |
|  | Ring a Bell campaign | Aimed to raise mental health awareness for K-8 students.  Implemented as of 2015 |
|  | Organizational culture change in the healthcare system | Aimed to facilitate adaptation of recovery model, no other details provided.  Implemented as of 2015 |
|  | Monitoring instruments | 2015 Community Addiction and Mental Health Services Client Satisfaction Survey: Primarily assessed services user satisfaction. Results indicated that the majority (~87%) of clients are satisfied with the services received |
| Goal 7: Improving populational mental health | Youth engagement process | Aimed to engage youth in community-based activities that promote resiliency and positive mental health.  Implemented as of 2015 |
|  | Expansion of suicide prevention strategies | No details provided  Implemented as of 2015 |
|  | Introduction of standardized assessment tools for mothers at-risk for mental illness | Implemented as of 2015 |
|  | Healthy Toddler Assessment | Designed for all families with 18-month-old children, this included a depression assessment component for parents.  Implemented as of 2015 |
|  | Eligibility criteria adjustment for Department of Education’s Attachment Program | Aimed to increase referral from Department of Health. The Attachment Program aimed to provide information and modelling to promote healthy, secure attachment between parents and child.  Implemented as of 2015 |
|  | Establishment of various campus mental health champion in 3 of the province's universities | Implemented as of 2015 |
|  | Wellness Movement | A social marketing initiative aimed to promote wellness in various settings.  Implemented as of 2015 |
|  | Monitoring instrument | 2014-2015 Elementary Student Wellness Survey: Assessed various mental fitness indicators that reduce vulnerabilities to future mental health issues. The 2015 report briefly described the distribution of the overall level of mental fitness amongst grade 4-5 students and no further findings were disseminated |

*Note.* Sources: (6; 7; 8; 9)
