# Supplementary material for: New Brunswick’s mental health action plan: A quantitative exploration of program efficacy in children and youth using the Canadian Community Health Survey
Source: PLoS One. 2024 Jun 7;19(6):e0301008. doi: 10.1371/journal.pone.0301008 (PMC11161078; doi:10.1371/journal.pone.0301008)
Supplement: S3 Table — (DOCX) [file pone.0301008.s007.docx]

| **S3 Table** |  |  |  |  |  | |  |
| --- | --- | --- | --- | --- | --- | --- | --- |
| *Block Regression Results when using the 2005 CCHS* | | | | | | | |
|  | Unstandardized *b /* Linearized Standard Error / 95% CI | | | | | | |
|  | Block 1 | | | Block 2 | | | |
| ***Model 1 (Sense of Belonging; N = 446)*** | | | | | | | |
| Constant | 1.71/0.56 | ^**^ | [0.62, 2.81] | 1.82/0.57 | ^**^ | | [0.71, 2.93] |
| Sex | 0.10/0.09 |  | [-0.07, 0.26] | 0.09/0.09 |  | | [-0.07, 0.26] |
| Marital Status | 0.35/0.52 |  | [-0.67, 1.36] | 0.29/0.51 |  | | [-0.72, 1.30] |
| Dwelling Ownership | 0.18/0.15 |  | [-0.10, 0.47] | 0.19/0.15 |  | | [-0.10, 0.48] |
| Self-rated Physical Health | 0.13/0.05 | ^*^ | [0.03, 0.23] | 0.12/0.05 | ^*^ | | [0.01, 0.22] |
| Household Income | -0.03/0.04 |  | [-0.11, 0.04] | -0.03/0.04 |  | | [-0.11, 0.04] |
| Household Size | 0.12/0.05 | ^*^ | [0.01, 0.22] | 0.11/0.05 | ^*^ | | [0.01, 0.22] |
| Visible Minority Status | 0.00/0.19 |  | [-0.37, 0.37] | 0.01/0.19 |  | | [-0.35, 0.38] |
| Vulnerable Population Status |  |  |  | -0.16/0.16 |  | | [-0.48, 0.16] |
| ***Model 2 (Mental Health Service Utilization; N = 450)*** | | | | | | | |
| Constant | 2.76/1.16 | ^*^ | [0.48, 5.05] | 1.52/1.29 |  | | [-1.01, 4.04] |
| Sex | -0.30/0.17 | ^†^ | [-0.63, 0.03] | -0.27/0.16 | ^†^ | | [-0.58, 0.04] |
| Marital Status | -1.05/0.96 |  | [-2.93, 0.84] | -0.44/1.21 |  | | [-2.82, 1.94] |
| Dwelling Ownership | 0.23/0.15 |  | [-0.07, 0.53] | 0.15/0.12 |  | | [-0.09, 0.39] |
| Self-rated Physical Health | -0.31/0.14 | ^*^ | [-0.59, -0.03] | -0.18/0.13 |  | | [-0.44, 0.08] |
| Household Income | 0.11/0.07 |  | [-0.04, 0.25] | 0.11/0.07 |  | | [-0.04, 0.25] |
| Household Size | -0.17/0.11 |  | [-0.39, 0.04] | -0.15/0.10 |  | | [-0.34, 0.05] |
| Visible Minority Status | -0.32/0.27 |  | [-0.85, 0.22] | -0.39/0.29 |  | | [-0.95, 0.17] |
| Vulnerable Population Status |  |  |  | 1.80/0.84 | ^*^ | | [0.14, 3.46] |
| ***Model 3 (Satisfaction with Life; N = 450)*** | | | | | | | |
| Constant | 2.65/0.43 | ^***^ | [1.80, 3.49] | 2.85/0.42 | ^***^ | [2.03, 3.67] | |
| Sex | 0.04/0.07 |  | [-0.09, 0.17] | 0.04/0.07 |  | [-0.09, 0.17] | |
| Marital Status | 0.51/0.42 |  | [-0.31, 1.34] | 0.41/0.41 |  | [-0.39, 1.22] | |
| Dwelling Ownership | 0.16/0.12 |  | [-0.08, 0.40] | 0.18/0.12 |  | [-0.06, 0.41] | |
| Self-rated Physical Health | 0.23/0.04 | ^***^ | [0.15, 0.31] | 0.21/0.04 | ^***^ | [0.14, 0.28] | |
| Household Income | 0.03/0.03 |  | [-0.04, 0.09] | 0.03/0.03 |  | [-0.03, 0.09] | |
| Household Size | 0.02/0.04 |  | [-0.06, 0.10] | 0.02/0.04 |  | [-0.06, 0.10] | |
| Visible Minority Status | 0.03/0.15 |  | [-0.27, 0.33] | 0.04/0.15 |  | [-0.26, 0.34] | |
| Vulnerable Population Status |  |  |  | -0.29/0.14 | ^*^ | [-0.57, -0.02] | |
| ***Model 4 (Life Stress; N = 253)*** | | | | | | | |
| Constant | 2.08/0.58 | ^***^ | [0.93, 3.23] | 2.62/0.58 | ^***^ | [1.47, 3.77] | |
| Sex | 0.33/0.14 | ^*^ | [0.06, 0.61] | 0.34/0.13 | ^*^ | [0.08, 0.60] | |
| Marital Status | 0.88/0.49 | ^†^ | [-0.10, 1.85] | 0.60/0.46 |  | [-0.32, 1.51] | |
| Dwelling Ownership | -0.22/0.22 |  | [-0.66, 0.22] | -0.18/0.22 |  | [-0.60, 0.24] | |
| Self-rated Physical Health | 0.06/0.09 |  | [-0.12, 0.24] | 0.00/0.09 |  | [-0.18, 0.17] | |
| Household Income | -0.12/0.06 | ^†^ | [-0.25, 0.00] | -0.11/0.06 | ^†^ | [-0.23, 0.01] | |
| Household Size | 0.21/0.08 | ^**^ | [0.06, 0.36] | 0.20/0.07 | ^**^ | [0.06, 0.34] | |
| Visible Minority Status | -0.10/0.27 |  | [-0.63, 0.43] | -0.10/0.27 |  | [-0.63, 0.43] | |
| Vulnerable Population Status |  |  |  | -0.77/0.16 | ^***^ | [-1.08, -0.46] | |
| *Note.* Vulnerable Population Status = Youths who identified as having a mood or anxiety disorder or rated their mental health as fair or poor; CI = Confidence Interval | | | | | | | |
| ^†^ *p* < .10; ^*^ *p* < .05; ^**^ *p* < .01; ^***^ *p* < .001 | | | | | | | |
