# Supplementary material for: New Brunswick’s mental health action plan: A quantitative exploration of program efficacy in children and youth using the Canadian Community Health Survey
Source: PLoS One. 2024 Jun 7;19(6):e0301008. doi: 10.1371/journal.pone.0301008 (PMC11161078; doi:10.1371/journal.pone.0301008)
Supplement: S4 Table — (DOCX) [file pone.0301008.s008.docx]

| **S4 Table** |  |  |  |  |  |  |
| --- | --- | --- | --- | --- | --- | --- |
| *Block Regression Results when using the 2007-2008 CCHS* | | | | | | |
|  | Unstandardized *b /* Linearized Standard Error / 95% CI | | | | | |
|  | Block 1 | | | Block 2 | | |
| ***Model 1 (Sense of Belonging; N = 369)*** | | | | | | |
| Constant | 1.42/0.68 | ^*^ | [0.08, 2.76] | 1.59/0.67 | ^*^ | [0.28, 2.91] |
| Sex | 0.00/0.11 |  | [-0.23, 0.22] | -0.03/0.11 |  | [-0.26, 0.19] |
| Marital Status | 0.58/0.66 |  | [-0.71, 1.87] | 0.66/0.62 |  | [-0.56, 1.89] |
| Dwelling Ownership | -0.07/0.18 |  | [-0.43, 0.29] | -0.12/0.19 |  | [-0.50, 0.26] |
| Self-rated Physical Health | 0.10/0.06 |  | [-0.02, 0.22] | 0.06/0.06 |  | [-0.06, 0.18] |
| Household Income | 0.04/0.07 |  | [-0.09, 0.16] | 0.04/0.06 |  | [-0.09, 0.17] |
| Household Size | 0.12/0.09 |  | [-0.06, 0.29] | 0.11/0.09 |  | [-0.06, 0.28] |
| Visible Minority Status | -0.05/0.38 |  | [-0.80, 0.71] | 0.01/0.33 |  | [-0.65, 0.66] |
| Vulnerable Population Status |  |  |  | -0.34/0.21 |  | [-0.76, 0.08] |
| ***Model 2 (Mental Health Service Utilization; N = 373)*** | | | | | | |
| Constant | 1.42/0.78 | ^†^ | [-0.11, 2.95] | 0.49/0.47 |  | [-0.44, 1.42] |
| Sex | -0.29/0.17 | ^†^ | [-0.63, 0.04] | -0.14/0.14 |  | [-0.41, 0.13] |
| Marital Status | 0.55/0.68 |  | [-0.79, 1.89] | 0.10/0.40 |  | [-0.69, 0.89] |
| Dwelling Ownership | 0.30/0.16 | ^†^ | [-0.01, 0.61] | 0.58/0.24 | ^*^ | [0.11, 1.05] |
| Self-rated Physical Health | -0.26/0.15 | ^†^ | [-0.57, 0.04] | -0.07/0.11 |  | [-0.28, 0.14] |
| Household Income | 0.06/0.07 |  | [-0.08, 0.20] | 0.03/0.06 |  | [-0.09, 0.16] |
| Household Size | -0.26/0.11 | ^*^ | [-0.48, -0.04] | -0.20/0.08 | ^*^ | [-0.36, -0.04] |
| Visible Minority Status | 1.52/0.95 |  | [-0.35, 3.40] | 1.24/0.68 | ^†^ | [-0.11, 2.58] |
| Vulnerable Population Status |  |  |  | 1.83/0.55 | ^**^ | [0.75, 2.91] |
| ***Model 3 (Satisfaction with Life; N = 373)*** | | | | | | |
| Constant | 3.90/0.28 | ^***^ | [3.34, 4.46] | 3.98/0.28 | ^***^ | [3.42, 4.54] |
| Sex | 0.02/0.07 |  | [-0.13, 0.16] | 0.00/0.07 |  | [-0.14, 0.15] |
| Marital Status | -0.60/0.24 | ^*^ | [-1.07, -0.12] | -0.56/0.24 | ^*^ | [-1.04, -0.07] |
| Dwelling Ownership | 0.11/0.10 |  | [-0.08, 0.31] | 0.09/0.10 |  | [-0.11, 0.29] |
| Self-rated Physical Health | 0.23/0.05 | ^***^ | [0.13, 0.33] | 0.21/0.05 | ^***^ | [0.12, 0.30] |
| Household Income | -0.01/0.03 |  | [-0.07, 0.06] | 0.00/0.03 |  | [-0.07, 0.06] |
| Household Size | 0.03/0.04 |  | [-0.05, 0.12] | 0.03/0.04 |  | [-0.05, 0.11] |
| Visible Minority Status | 0.08/0.13 |  | [-0.16, 0.33] | 0.11/0.12 |  | [-0.12, 0.34] |
| Vulnerable Population Status |  |  |  | -0.17/0.12 |  | [-0.40, 0.07] |
| ***Model 4 (Life Stress; N = 252)*** | | | | | | |
| Constant | 2.45/0.38 | ^***^ | [1.70, 3.21] | 2.60/0.33 | ^***^ | [1.95, 3.25] |
| Sex | 0.38/0.14 | ^**^ | [0.10, 0.66] | 0.32/0.13 | ^*^ | [0.06, 0.58] |
| Marital Status | -0.72/0.31 | ^*^ | [-1.33, -0.10] | -0.51/0.28 | ^†^ | [-1.05, 0.04] |
| Dwelling Ownership | 0.29/0.22 |  | [-0.14, 0.73] | 0.20/0.18 |  | [-0.16, 0.57] |
| Self-rated Physical Health | 0.18/0.10 | ^†^ | [-0.01, 0.37] | 0.14/0.08 | ^†^ | [-0.01, 0.30] |
| Household Income | -0.04/0.08 |  | [-0.19, 0.10] | -0.06/0.07 |  | [-0.20, 0.07] |
| Household Size | 0.16/0.09 | ^†^ | [-0.01, 0.33] | 0.19/0.08 | ^*^ | [0.03, 0.34] |
| Visible Minority Status | 0.10/0.18 |  | [-0.26, 0.46] | 0.27/0.19 |  | [-0.11, 0.65] |
| Vulnerable Population Status |  |  |  | -0.54/0.15 | ^***^ | [-0.83, -0.25] |
| *Note.* Vulnerable Population Status = Youths who identified as having a mood or anxiety disorder or rated their mental health as fair or poor; CI = Confidence Interval | | | | | | |
| ^†^ *p* < .10; ^*^ *p* < .05; ^**^ *p* < .01; ^***^ *p* < .001 | | | | | | |
