# Supplementary material for: New Brunswick’s mental health action plan: A quantitative exploration of program efficacy in children and youth using the Canadian Community Health Survey
Source: PLoS One. 2024 Jun 7;19(6):e0301008. doi: 10.1371/journal.pone.0301008 (PMC11161078; doi:10.1371/journal.pone.0301008)
Supplement: S5 Table — (DOCX) [file pone.0301008.s009.docx]

| **S5 Table** | |  | |  | |  |  |  |  |
| --- | --- | --- | --- | --- | --- | --- | --- | --- | --- |
| *Block Regression Results when using the 2009-2010 CCHS* | | | | | | | | | |
|  | | Unstandardized *b /* Linearized Standard Error / 95% CI | | | | | | | |
|  | | Block 1 | | | | | Block 2 | | |
| ***Model 1 (Sense of Belonging; N = 335)*** | | | | | | | | | |
| Constant | | 1.78/0.55 | | ^**^ | | [0.70, 2.87] | 2.04/0.47 | ^***^ | [1.12, 2.96] |
| Sex | | -0.12/0.09 | |  | | [-0.29, 0.06] | -0.12/0.09 |  | [-0.29, 0.06] |
| Marital Status | | -0.26/0.39 | |  | | [-1.02, 0.49] | -0.38/0.41 |  | [-1.19, 0.43] |
| Dwelling Ownership | | 0.02/0.17 | |  | | [-0.31, 0.36] | 0.04/0.16 |  | [-0.28, 0.36] |
| Self-rated Physical Health | | 0.26/0.10 | | ^**^ | | [0.07, 0.44] | 0.24/0.08 | ^**^ | [0.08, 0.39] |
| Household Income | | 0.04/0.03 | |  | | [-0.02, 0.11] | 0.04/0.03 |  | [-0.02, 0.10] |
| Household Size | | 0.12/0.04 | | ^**^ | | [0.03, 0.20] | 0.11/0.04 | ^**^ | [0.03, 0.19] |
| Visible Minority Status | | -0.19/0.12 | |  | | [-0.43, 0.06] | -0.18/0.13 |  | [-0.43, 0.07] |
| Vulnerable Population Status | |  | |  | |  | -0.18/0.23 |  | [-0.63, 0.26] |
| ***Model 2 (Mental Health Service Utilization; N = 337)*** | | | | | | | | | |
| Constant | | 1.65/0.60 | | ^**^ | [0.46, 2.83] | | 0.91/0.89 |  | [-0.85, 2.66] |
| Sex | | -0.19/0.18 | |  | [-0.54, 0.16] | | -0.19/0.18 |  | [-0.54, 0.15] |
| Marital Status | | 0.56/0.37 | |  | [-0.16, 1.29] | | 0.90/0.33 | ^**^ | [0.24, 1.55] |
| Dwelling Ownership | | 0.25/0.30 | |  | [-0.33, 0.84] | | 0.21/0.29 |  | [-0.37, 0.78] |
| Self-rated Physical Health | | -0.21/0.11 | | ^*^ | [-0.43, 0.00] | | -0.15/0.11 |  | [-0.37, 0.06] |
| Household Income | | -0.13/0.06 | | ^*^ | [-0.26, 0.00] | | -0.11/0.07 |  | [-0.25, 0.03] |
| Household Size | | -0.16/0.10 | |  | [-0.36, 0.04] | | -0.14/0.11 |  | [-0.35, 0.08] |
| Visible Minority Status | | -0.23/0.10 | | ^*^ | [-0.43, -0.03] | | -0.24/0.11 | ^*^ | [-0.45, -0.03] |
| Vulnerable Population Status | |  | |  |  | | 0.54/0.37 |  | [-0.20, 1.27] |
| ***Model 3 (Satisfaction with Life; N = 337)*** | | | | | | | | | |
| Constant | 3.87/0.29 | | ^***^ | | [3.30, 4.44] | | 4.20/0.35 | ^***^ | [3.51, 4.89] |
| Sex | 0.01/0.08 | |  | | [-0.15, 0.16] | | 0.01/0.08 |  | [-0.15, 0.16] |
| Marital Status | 0.00/0.26 | |  | | [-0.51, 0.52] | | -0.14/0.27 |  | [-0.69, 0.40] |
| Dwelling Ownership | 0.08/0.20 | |  | | [-0.30, 0.47] | | 0.10/0.20 |  | [-0.28, 0.49] |
| Self-rated Physical Health | 0.21/0.04 | | ^***^ | | [0.13, 0.30] | | 0.19/0.05 | ^***^ | [0.09, 0.28] |
| Household Income | -0.01/0.03 | |  | | [-0.07, 0.05] | | -0.02/0.03 |  | [-0.08, 0.04] |
| Household Size | -0.06/0.05 | |  | | [-0.16, 0.04] | | -0.07/0.05 |  | [-0.17, 0.03] |
| Visible Minority Status | -0.35/0.15 | | ^*^ | | [-0.64, -0.06] | | -0.35/0.15 | ^*^ | [-0.64, -0.06] |
| Vulnerable Population Status |  | |  | |  | | -0.24/0.16 |  | [-0.56, 0.08] |
| ***Model 4 (Life Stress; N = 350)*** | | | | | | | | | |
| Constant | 1.73/0.58 | | ^**^ | | [0.59, 2.86] | | 2.20/0.56 | ^***^ | [1.09, 3.30] |
| Sex | 0.09/0.13 | |  | | [-0.16, 0.35] | | 0.08/0.13 |  | [-0.17, 0.33] |
| Marital Status | 0.47/0.30 | |  | | [-0.11, 1.06] | | 0.28/0.38 |  | [-0.47, 1.03] |
| Dwelling Ownership | -0.30/0.25 | |  | | [-0.79, 0.20] | | -0.25/0.25 |  | [-0.73, 0.23] |
| Self-rated Physical Health | 0.31/0.12 | | ^*^ | | [0.08, 0.55] | | 0.27/0.11 | ^*^ | [0.06, 0.48] |
| Household Income | 0.08/0.06 | |  | | [-0.03, 0.19] | | 0.07/0.06 |  | [-0.04, 0.18] |
| Household Size | 0.01/0.07 | |  | | [-0.14, 0.15] | | -0.01/0.07 |  | [-0.15, 0.13] |
| Visible Minority Status | -0.25/0.27 | |  | | [-0.78, 0.28] | | -0.19/0.30 |  | [-0.78, 0.41] |
| Vulnerable Population Status |  | |  | |  | | -0.35/0.21 |  | [-0.77, 0.07] |
| *Note.* Vulnerable Population Status = Youths who identified as having a mood or anxiety disorder or rated their mental health as fair or poor; CI = Confidence Interval | | | | | | | | | |
| ^†^ *p* < .10; ^*^ *p* < .05; ^**^ *p* < .01; ^***^ *p* < .001 | | | | | | | | | |
