# Supplementary material for: New Brunswick’s mental health action plan: A quantitative exploration of program efficacy in children and youth using the Canadian Community Health Survey
Source: PLoS One. 2024 Jun 7;19(6):e0301008. doi: 10.1371/journal.pone.0301008 (PMC11161078; doi:10.1371/journal.pone.0301008)
Supplement: S6 Table — (DOCX) [file pone.0301008.s010.docx]

| **S6 Table** |  |  |  |  |  |  |
| --- | --- | --- | --- | --- | --- | --- |
| *Block Regression Results when using the 2011-2012 CCHS* | | | | | | |
|  | Unstandardized *b /* Linearized Standard Error / 95% CI | | | | | |
|  | Block 1 | | | Block 2 | | |
| ***Model 1 (Sense of Belonging; N = 423)*** | | | | | | |
| Constant | 1.35/0.71 | ^†^ | [-0.05, 2.75] | 1.76/0.55 | ^**^ | [0.69, 2.84] |
| Sex | -0.02/0.10 |  | [-0.21, 0.18] | -0.05/0.10 |  | [-0.24, 0.14] |
| Marital Status | 0.49/0.61 |  | [-0.70, 1.69] | 0.37/0.48 |  | [-0.58, 1.32] |
| Dwelling Ownership | 0.12/0.16 |  | [-0.21, 0.44] | 0.07/0.16 |  | [-0.24, 0.38] |
| Self-rated Physical Health | 0.16/0.08 | ^†^ | [0.00, 0.32] | 0.12/0.07 |  | [-0.03, 0.26] |
| Household Income | 0.06/0.04 | ^†^ | [-0.01, 0.14] | 0.06/0.04 | ^†^ | [-0.01, 0.13] |
| Household Size | 0.05/0.06 |  | [-0.07, 0.17] | 0.05/0.05 |  | [-0.06, 0.16] |
| Visible Minority Status | 0.32/0.18 | ^†^ | [-0.02, 0.67] | 0.35/0.17 | ^*^ | [0.02, 0.68] |
| Vulnerable Population Status |  |  |  | -0.51/0.19 | ^**^ | [-0.89, -0.14] |
| ***Model 2 (Mental Health Service Utilization; N = 430)*** | | | | | | |
| Constant | -0.75/0.44 | ^†^ | [-1.61, 0.11] | 1.07/1.64 |  | [-2.15, 4.29] |
| Sex | 0.23/1.44 |  | [-2.59, 3.05] | -0.54/0.41 |  | [-1.34, 0.26] |
| Marital Status | -1.79/1.06 | ^†^ | [-3.87, 0.28] | 0.96/0.89 |  | [-0.79, 2.70] |
| Dwelling Ownership | -0.07/0.45 |  | [-0.95, 0.80] | -1.52/0.98 |  | [-3.45, 0.42] |
| Self-rated Physical Health | 0.22/0.14 |  | [-0.06, 0.50] | 0.20/0.37 |  | [-0.53, 0.92] |
| Household Income | -0.36/0.27 |  | [-0.88, 0.16] | 0.23/0.13 | ^†^ | [-0.03, 0.48] |
| Household Size | -0.28/0.74 |  | [-1.73, 1.18] | -0.36/0.23 |  | [-0.82, 0.10] |
| Visible Minority Status | 3.50/2.65 |  | [-1.71, 8.71] | -0.43/0.80 |  | [-2.01, 1.14] |
| Vulnerable Population Status |  |  |  | 3.01/0.96 | ^**^ | [1.13, 4.90] |
| ***Model 3 (Satisfaction with Life; N = 428)*** | | | | | | |
| Constant | -0.75/0.44 | ^†^ | [-1.61, 0.11] | 1.07/1.64 |  | [-2.15, 4.29] |
| Sex | 0.23/1.44 |  | [-2.59, 3.05] | -0.54/0.41 |  | [-1.34, 0.26] |
| Marital Status | -1.79/1.06 | ^†^ | [-3.87, 0.28] | 0.96/0.89 |  | [-0.79, 2.70] |
| Dwelling Ownership | -0.07/0.45 |  | [-0.95, 0.80] | -1.52/0.98 |  | [-3.45, 0.42] |
| Self-rated Physical Health | 0.22/0.14 |  | [-0.06, 0.50] | 0.20/0.37 |  | [-0.53, 0.92] |
| Household Income | -0.36/0.27 |  | [-0.88, 0.16] | 0.23/0.13 | ^†^ | [-0.03, 0.48] |
| Household Size | -0.28/0.74 |  | [-1.73, 1.18] | -0.36/0.23 |  | [-0.82, 0.10] |
| Visible Minority Status | 3.50/2.65 |  | [-1.71, 8.71] | -0.43/0.80 |  | [-2.01, 1.14] |
| Vulnerable Population Status |  |  |  | 3.01/0.96 | ^**^ | [1.13, 4.90] |
| ***Model 4 (Life Stress; N = 440)*** | | | | | | |
| Constant | 2.14/0.59 | ^***^ | [0.98, 3.30] | 2.71/0.49 | ^***^ | [1.76, 3.66] |
| Sex | 0.24/0.11 | ^*^ | [0.02, 0.46] | 0.20/0.11 | ^†^ | [-0.02, 0.41] |
| Marital Status | -0.14/0.37 |  | [-0.87, 0.58] | -0.30/0.29 |  | [-0.88, 0.28] |
| Dwelling Ownership | 0.42/0.14 | ^**^ | [0.14, 0.70] | 0.35/0.13 | ^**^ | [0.10, 0.61] |
| Self-rated Physical Health | 0.18/0.08 | ^*^ | [0.02, 0.34] | 0.13/0.07 | ^†^ | [-0.01, 0.27] |
| Household Income | -0.03/0.05 |  | [-0.11, 0.06] | -0.03/0.05 |  | [-0.12, 0.06] |
| Household Size | 0.12/0.07 | ^†^ | [-0.01, 0.26] | 0.12/0.06 | ^†^ | [-0.01, 0.24] |
| Visible Minority Status | 0.21/0.19 |  | [-0.16, 0.58] | 0.23/0.19 |  | [-0.14, 0.61] |
| Vulnerable Population Status |  |  |  | -0.70/0.19 | ^***^ | [-1.07, -0.33] |
| *Note.* Vulnerable Population Status = Youths who identified as having a mood or anxiety disorder or rated their mental health as fair or poor; CI = Confidence Interval | | | | | | |
| ^†^ *p* < .10; ^*^ *p* < .05; ^**^ *p* < .01; ^***^ *p* < .001 | | | | | | |
