# Supplementary material for: New Brunswick’s mental health action plan: A quantitative exploration of program efficacy in children and youth using the Canadian Community Health Survey
Source: PLoS One. 2024 Jun 7;19(6):e0301008. doi: 10.1371/journal.pone.0301008 (PMC11161078; doi:10.1371/journal.pone.0301008)
Supplement: S7 Table — (DOCX) [file pone.0301008.s011.docx]

| **S7Table** |  |  |  |  |  |  |
| --- | --- | --- | --- | --- | --- | --- |
| *Block Regression Results when using the 2015-2016 CCHS* | | | | | | |
|  | Unstandardized *b /* Linearized Standard Error / 95% CI | | | | | |
|  | Block 1 | | | Block 2 | | |
| ***Model 1 (Sense of Belonging; N = 288)*** | | | | | | |
| Constant | 1.26/0.32 | ^***^ | [-0.05, 2.75] | 1.50/0.29 | ^***^ | [-0.05, 2.75] |
| Sex | 0.14/0.14 |  | [-0.21, 0.18] | 0.10/0.14 |  | [-0.21, 0.18] |
| Marital Status | 0.61/0.26 | ^*^ | [-0.70, 1.69] | 0.76/0.30 | ^*^ | [-0.70, 1.69] |
| Dwelling Ownership | 0.04/0.15 |  | [-0.21, 0.44] | 0.00/0.14 |  | [-0.21, 0.44] |
| Self-rated Physical Health | 0.13/0.09 |  | [0.00, 0.32] | 0.09/0.08 |  | [0.00, 0.32] |
| Household Income | -0.01/0.05 |  | [-0.01, 0.14] | -0.02/0.04 |  | [-0.01, 0.14] |
| Household Size | 0.15/0.09 | ^†^ | [-0.07, 0.17] | 0.13/0.09 |  | [-0.07, 0.17] |
| Visible Minority Status | 0.06/0.23 |  | [-0.02, 0.67] | -0.03/0.24 |  | [-0.02, 0.67] |
| Vulnerable Population Status |  |  |  | -0.39/0.15 | ^**^ | [-0.05, 2.75] |
| ***Model 2 (Mental Health Service Utilization; N = 287)*** | | | | | | |
| Constant | 2.26/0.79 | ^**^ | [-0.05, 2.75] | 1.11/0.64 | ^†^ | [-0.05, 2.75] |
| Sex | -0.18/0.18 |  | [-0.21, 0.18] | 0.00/0.15 |  | [-0.21, 0.18] |
| Marital Status | 1.79/0.65 | ^**^ | [-0.70, 1.69] | 1.07/0.56 | ^†^ | [-0.70, 1.69] |
| Dwelling Ownership | -0.65/0.43 |  | [-0.21, 0.44] | -0.48/0.41 |  | [-0.21, 0.44] |
| Self-rated Physical Health | -0.39/0.16 | ^*^ | [0.00, 0.32] | -0.21/0.13 |  | [0.00, 0.32] |
| Household Income | -0.04/0.08 |  | [-0.01, 0.14] | 0.02/0.08 |  | [-0.01, 0.14] |
| Household Size | -0.30/0.14 | ^*^ | [-0.07, 0.17] | -0.21/0.12 | ^†^ | [-0.07, 0.17] |
| Visible Minority Status | -0.78/0.35 | ^*^ | [-0.02, 0.67] | -0.39/0.29 |  | [-0.02, 0.67] |
| Vulnerable Population Status |  |  |  | 1.89/0.43 | ^***^ | [-0.05, 2.75] |
| ***Model 3 (Satisfaction with Life; N = 287)*** | | | | | | |
| Constant | 2.69/0.32 | ^***^ | [-0.05, 2.75] | 2.93/0.28 | ^***^ | [-0.05, 2.75] |
| Sex | 0.02/0.09 |  | [-0.21, 0.18] | -0.02/0.09 |  | [-0.21, 0.18] |
| Marital Status | 0.26/0.22 |  | [-0.70, 1.69] | 0.41/0.23 | ^†^ | [-0.70, 1.69] |
| Dwelling Ownership | -0.02/0.14 |  | [-0.21, 0.44] | -0.05/0.15 |  | [-0.21, 0.44] |
| Self-rated Physical Health | 0.33/0.07 | ^***^ | [0.00, 0.32] | 0.29/0.06 | ^***^ | [0.00, 0.32] |
| Household Income | 0.01/0.05 |  | [-0.01, 0.14] | 0.00/0.05 |  | [-0.01, 0.14] |
| Household Size | 0.04/0.05 |  | [-0.07, 0.17] | 0.02/0.05 |  | [-0.07, 0.17] |
| Visible Minority Status | -0.12/0.18 |  | [-0.02, 0.67] | -0.20/0.18 |  | [-0.02, 0.67] |
| Vulnerable Population Status |  |  |  | -0.39/0.18 | ^*^ | [-0.05, 2.75] |
| ***Model 4 (Life Stress; N = 297)*** | | | | | | |
| Constant | 1.99/0.37 | ^***^ | [-0.05, 2.75] | 2.40/0.34 | ^***^ | [-0.05, 2.75] |
| Sex | 0.29/0.15 | ^†^ | [-0.21, 0.18] | 0.26/0.15 | ^†^ | [-0.21, 0.18] |
| Marital Status | 0.12/0.29 |  | [-0.70, 1.69] | 0.37/0.30 |  | [-0.70, 1.69] |
| Dwelling Ownership | 0.46/0.24 | ^†^ | [-0.21, 0.44] | 0.42/0.24 | ^†^ | [-0.21, 0.44] |
| Self-rated Physical Health | 0.25/0.10 | ^**^ | [0.00, 0.32] | 0.19/0.09 | ^*^ | [0.00, 0.32] |
| Household Income | -0.02/0.06 |  | [-0.01, 0.14] | -0.05/0.06 |  | [-0.01, 0.14] |
| Household Size | 0.00/0.09 |  | [-0.07, 0.17] | -0.03/0.09 |  | [-0.07, 0.17] |
| Visible Minority Status | 0.43/0.34 |  | [-0.02, 0.67] | 0.29/0.34 |  | [-0.02, 0.67] |
| Vulnerable Population Status |  |  |  | -0.58/0.23 | ^*^ | [-0.05, 2.75] |
| *Note.* Vulnerable Population Status = Youths who identified as having a mood or anxiety disorder or rated their mental health as fair or poor; CI = Confidence Interval | | | | | | |
| ^†^ *p* < .10; ^*^ *p* < .05; ^**^ *p* < .01; ^***^ *p* < .001 | | | | | | |
